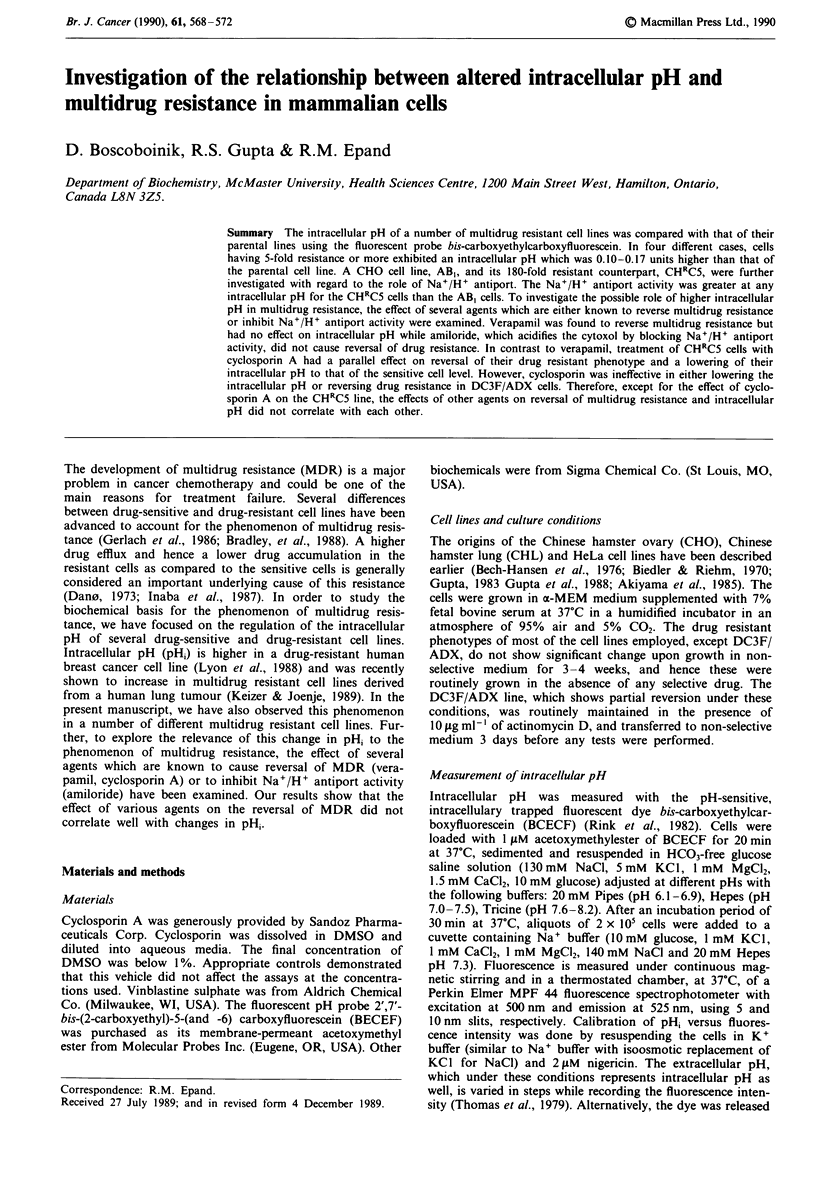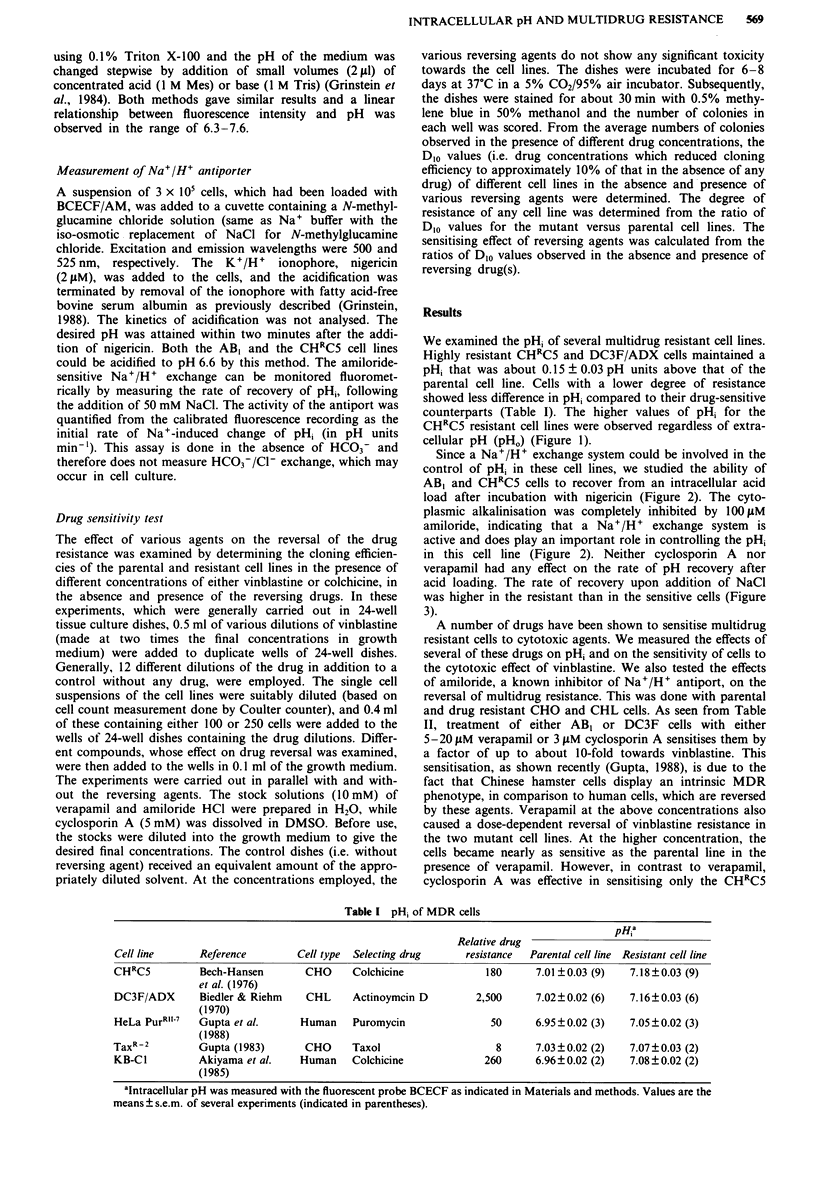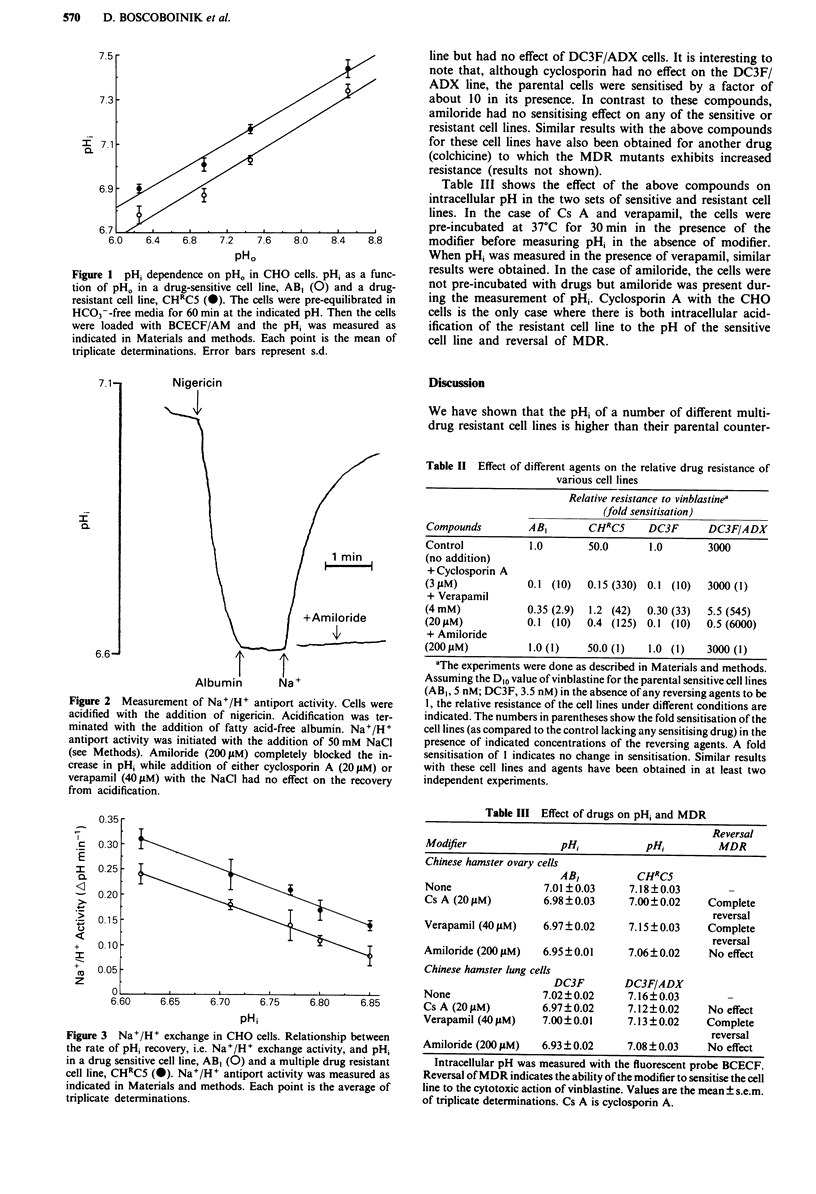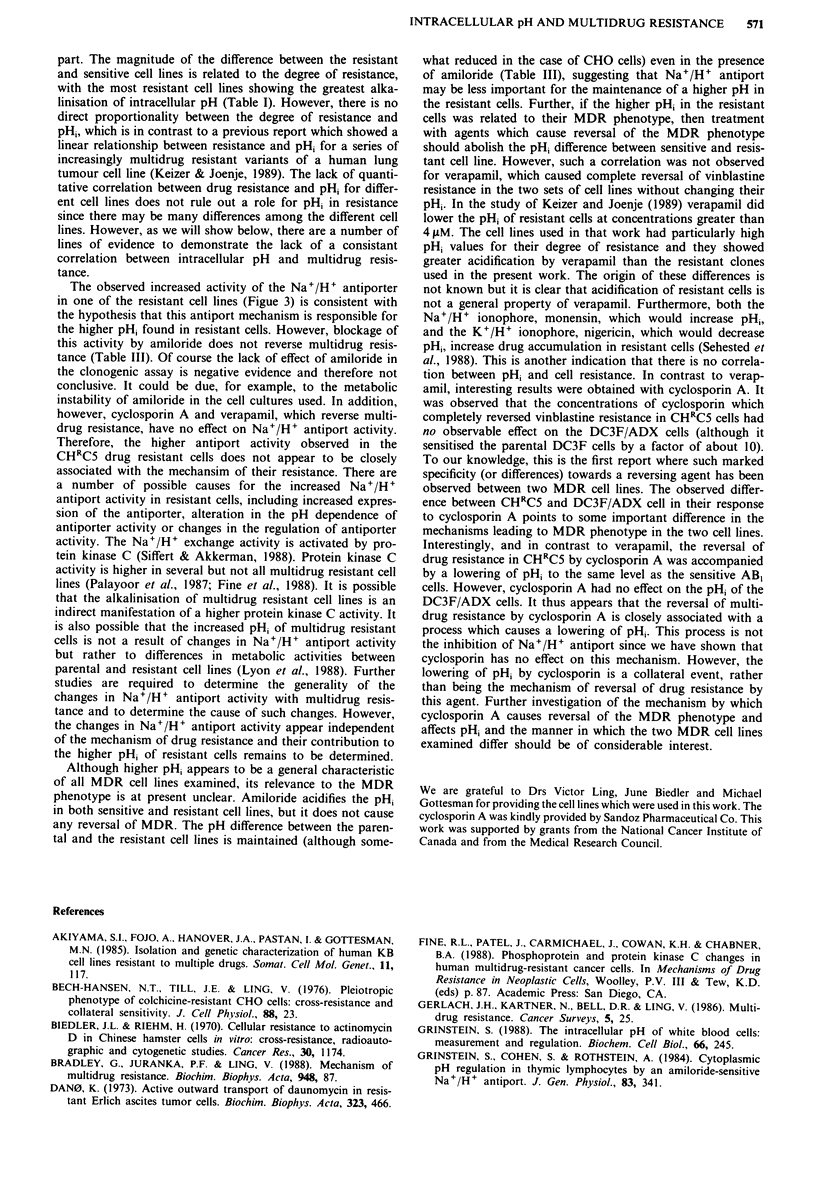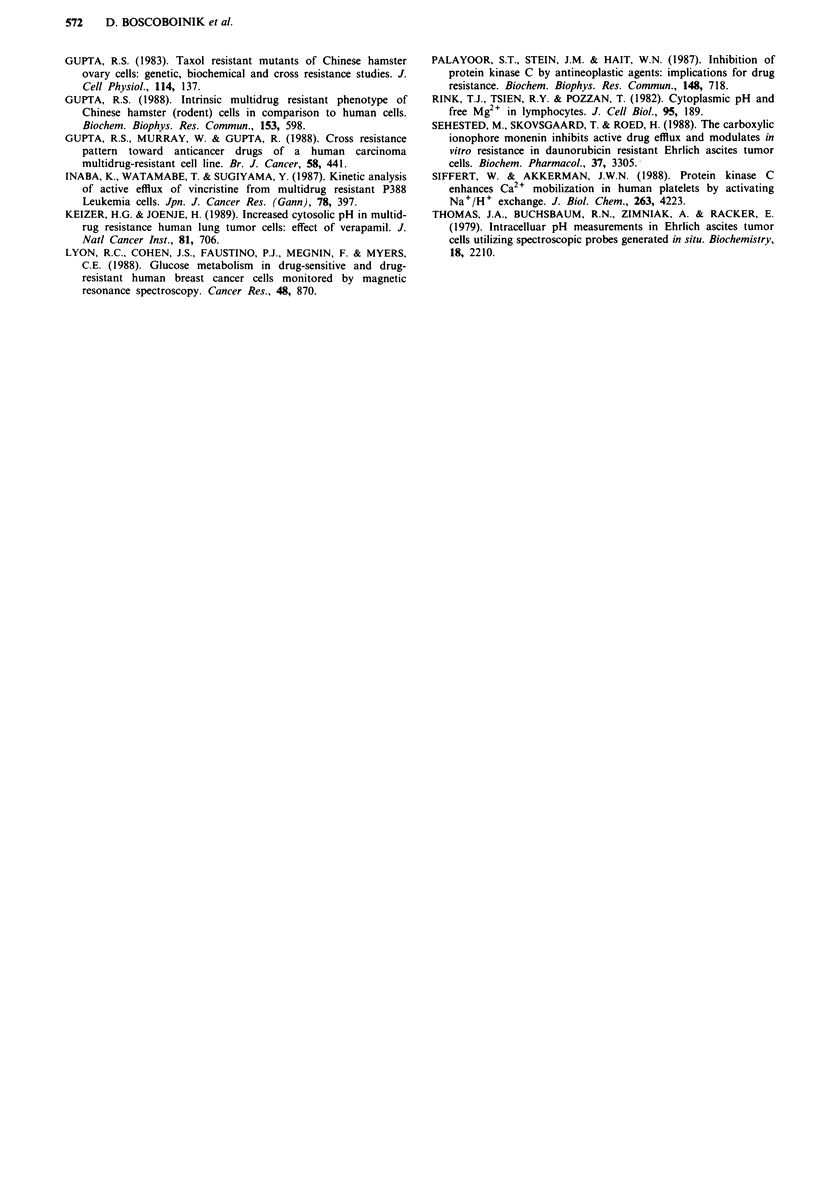# Investigation of the relationship between altered intracellular pH and multidrug resistance in mammalian cells.

**DOI:** 10.1038/bjc.1990.127

**Published:** 1990-04

**Authors:** D. Boscoboinik, R. S. Gupta, R. M. Epand

**Affiliations:** Department of Biochemistry, McMaster University, Hamilton, Ontario, Canada.

## Abstract

The intracellular pH of a number of multidrug resistant cell lines was compared with that of their parental lines using the fluorescent probe bis-carboxyethylcarboxyfluorescein. In four different cases, cells having 5-fold resistance or more exhibited an intracellular pH which was 0.10-0.17 units higher than that of the parental cell line. A CHO cell line, AB1, and its 180-fold resistant counterpart, CHRC5, were further investigated with regard to the role of Na+/H+ antiport. The Na+/H+ antiport activity was greater at any intracellular pH for the CHRC5 cells than the AB1 cells. To investigate the possible role of higher intracellular pH in multidrug resistance, the effect of several agents which are either known to reverse multidrug resistance or inhibit Na+/H+ antiport activity were examined. Verapamil was found to reverse multidrug resistance but had no effect on intracellular pH while amiloride, which acidifies the cytoxol by blocking Na+/H+ antiport activity, did not cause reversal of drug resistance. In contrast to verapamil, treatment of CHRC5 cells with cyclosporin A had a parallel effect on reversal of their drug resistant phenotype and a lowering of their intracellular pH to that of the sensitive cell level. However, cyclosporin was ineffective in either lowering the intracellular pH or reversing drug resistance in DC3F/ADX cells. Therefore, except for the effect of cyclosporin A on the CHRC5 line, the effects of other agents on reversal of multidrug resistance and intracellular pH did not correlate with each other.


Br. J. Cancer (1990), 61, 568-572 © Macmillan Press Ltd., 1990

### Investigation of the relationship between altered intracellular pH and multidrug resistance in mammalian cells

D. Boscoboinik, R.S. Gupta & R.M. Epand

*Department of Biochemistry, McMaster University, Health Sciences Centre, 1200 Main Street West, Hamilton, Ontario, Canada L8N 3Z5.*

**Summary** The intracellular pH of a number of multidrug resistant cell lines was compared with that of their parental lines using the fluorescent probe *bis*-carboxyethylcarboxyfluorescein. In four different cases, cells having 5-fold resistance or more exhibited an intracellular pH which was 0.10-0.17 units higher than that of the parental cell line. A CHO cell line, $AB_1$, and its 180-fold resistant counterpart, $CH^RC5$, were further investigated with regard to the role of $Na^+/H^+$ antiport. The $Na^+/H^+$ antiport activity was greater at any intracellular pH for the $CH^RC5$ cells than the $AB_1$ cells. To investigate the possible role of higher intracellular pH in multidrug resistance, the effect of several agents which are either known to reverse multidrug resistance or inhibit $Na^+/H^+$ antiport activity were examined. Verapamil was found to reverse multidrug resistance but had no effect on intracellular pH while amiloride, which acidifies the cytoxol by blocking $Na^+/H^+$ antiport activity, did not cause reversal of drug resistance. In contrast to verapamil, treatment of $CH^RC5$ cells with cyclosporin A had a parallel effect on reversal of their drug resistant phenotype and a lowering of their intracellular pH to that of the sensitive cell level. However, cyclosporin was ineffective in either lowering the intracellular pH or reversing drug resistance in DC3F/ADX cells. Therefore, except for the effect of cyclosporin A on the $CH^RC5$ line, the effects of other agents on reversal of multidrug resistance and intracellular pH did not correlate with each other.

The development of multidrug resistance (MDR) is a major problem in cancer chemotherapy and could be one of the main reasons for treatment failure. Several differences between drug-sensitive and drug-resistant cell lines have been advanced to account for the phenomenon of multidrug resistance (Gerlach *et al.*, 1986; Bradley, *et al.*, 1988). A higher drug efflux and hence a lower drug accumulation in the resistant cells as compared to the sensitive cells is generally considered an important underlying cause of this resistance (Danø, 1973; Inaba *et al.*, 1987). In order to study the biochemical basis for the phenomenon of multidrug resistance, we have focused on the regulation of the intracellular pH of several drug-sensitive and drug-resistant cell lines. Intracellular pH ($pH_i$) is higher in a drug-resistant human breast cancer cell line (Lyon *et al.*, 1988) and was recently shown to increase in multidrug resistant cell lines derived from a human lung tumour (Keizer & Joenje, 1989). In the present manuscript, we have also observed this phenomenon in a number of different multidrug resistant cell lines. Further, to explore the relevance of this change in $pH_i$ to the phenomenon of multidrug resistance, the effect of several agents which are known to cause reversal of MDR (verapamil, cyclosporin A) or to inhibit $Na^+/H^+$ antiport activity (amiloride) have been examined. Our results show that the effect of various agents on the reversal of MDR did not correlate well with changes in $pH_i$.

### Materials and methods

### *Materials*

Cyclosporin A was generously provided by Sandoz Pharmaceuticals Corp. Cyclosporin was dissolved in DMSO and diluted into aqueous media. The final concentration of DMSO was below 1%. Appropriate controls demonstrated that this vehicle did not affect the assays at the concentrations used. Vinblastine sulphate was from Aldrich Chemical Co. (Milwaukee, WI, USA). The fluorescent pH probe 2′,7′-*bis*-(2-carboxyethyl)-5-(and -6) carboxyfluorescein (BECEF) was purchased as its membrane-permeant acetoxymethyl ester from Molecular Probes Inc. (Eugene, OR, USA). Other biochemicals were from Sigma Chemical Co. (St Louis, MO, USA).

### *Cell lines and culture conditions*

The origins of the Chinese hamster ovary (CHO), Chinese hamster lung (CHL) and HeLa cell lines have been described earlier (Bech-Hansen *et al.*, 1976; Biedler & Riehm, 1970; Gupta, 1983 Gupta *et al.*, 1988; Akiyama *et al.*, 1985). The cells were grown in α-MEM medium supplemented with 7% fetal bovine serum at 37°C in a humidified incubator in an atmosphere of 95% air and 5% $CO_2$. The drug resistant phenotypes of most of the cell lines employed, except DC3F/ADX, do not show significant change upon growth in non-selective medium for 3-4 weeks, and hence these were routinely grown in the absence of any selective drug. The DC3F/ADX line, which shows partial reversion under these conditions, was routinely maintained in the presence of 10 $\mu g\ ml^{-1}$ of actinomycin D, and transferred to non-selective medium 3 days before any tests were performed.

### *Measurement of intracellular pH*

Intracellular pH was measured with the pH-sensitive, intracellulary trapped fluorescent dye *bis*-carboxyethylcarboxyfluorescein (BCECF) (Rink *et al.*, 1982). Cells were loaded with 1 μM acetoxymethylester of BCECF for 20 min at 37°C, sedimented and resuspended in $HCO_3$-free glucose saline solution (130 mM NaCl, 5 mM KCl, 1 mM $MgCl_2$, 1.5 mM $CaCl_2$, 10 mM glucose) adjusted at different pHs with the following buffers: 20 mM Pipes (pH 6.1-6.9), Hepes (pH 7.0-7.5), Tricine (pH 7.6-8.2). After an incubation period of 30 min at 37°C, aliquots of $2 \times 10^5$ cells were added to a cuvette containing $Na^+$ buffer (10 mM glucose, 1 mM KCl, 1 mM $CaCl_2$, 1 mM $MgCl_2$, 140 mM NaCl and 20 mM Hepes pH 7.3). Fluorescence is measured under continuous magnetic stirring and in a thermostated chamber, at 37°C, of a Perkin Elmer MPF 44 fluorescence spectrophotometer with excitation at 500 nm and emission at 525 nm, using 5 and 10 nm slits, respectively. Calibration of $pH_i$ versus fluorescence intensity was done by resuspending the cells in $K^+$ buffer (similar to $Na^+$ buffer with isoosmotic replacement of KCl for NaCl) and 2 μM nigericin. The extracellular pH, which under these conditions represents intracellular pH as well, is varied in steps while recording the fluorescence intensity (Thomas *et al.*, 1979). Alternatively, the dye was released

Correspondence: R.M. Epand.
Received 27 July 1989; and in revised form 4 December 1989.

using 0.1% Triton X-100 and the pH of the medium was changed stepwise by addition of small volumes (2 μl) of concentrated acid (1 M Mes) or base (1 M Tris) (Grinstein *et al.*, 1984). Both methods gave similar results and a linear relationship between fluorescence intensity and pH was observed in the range of 6.3–7.6.

### *Measurement of $Na^+/H^+$ antiporter*

A suspension of $3 \times 10^5$ cells, which had been loaded with BCECF/AM, was added to a cuvette containing a *N*-methylglucamine chloride solution (same as $Na^+$ buffer with the iso-osmotic replacement of NaCl for *N*-methylglucamine chloride. Excitation and emission wavelengths were 500 and 525 nm, respectively. The $K^+/H^+$ ionophore, nigericin (2 μM), was added to the cells, and the acidification was terminated by removal of the ionophore with fatty acid-free bovine serum albumin as previously described (Grinstein, 1988). The kinetics of acidification was not analysed. The desired pH was attained within two minutes after the addition of nigericin. Both the $AB_1$ and the $CH^RC5$ cell lines could be acidified to pH 6.6 by this method. The amiloride-sensitive $Na^+/H^+$ exchange can be monitored fluorometrically by measuring the rate of recovery of $pH_i$, following the addition of 50 mM NaCl. The activity of the antiport was quantified from the calibrated fluorescence recording as the initial rate of $Na^+$-induced change of $pH_i$ (in pH units $min^{-1}$). This assay is done in the absence of $HCO_3^-$ and therefore does not measure $HCO_3^-/Cl^-$ exchange, which may occur in cell culture.

### *Drug sensitivity test*

The effect of various agents on the reversal of the drug resistance was examined by determining the cloning efficiencies of the parental and resistant cell lines in the presence of different concentrations of either vinblastine or colchicine, in the absence and presence of the reversing drugs. In these experiments, which were generally carried out in 24-well tissue culture dishes, 0.5 ml of various dilutions of vinblastine (made at two times the final concentrations in growth medium) were added to duplicate wells of 24-well dishes. Generally, 12 different dilutions of the drug in addition to a control without any drug, were employed. The single cell suspensions of the cell lines were suitably diluted (based on cell count measurement done by Coulter counter), and 0.4 ml of these containing either 100 or 250 cells were added to the wells of 24-well dishes containing the drug dilutions. Different compounds, whose effect on drug reversal was examined, were then added to the wells in 0.1 ml of the growth medium. The experiments were carried out in parallel with and without the reversing agents. The stock solutions (10 mM) of verapamil and amiloride HCl were prepared in $H_2O$, while cyclosporin A (5 mM) was dissolved in DMSO. Before use, the stocks were diluted into the growth medium to give the desired final concentrations. The control dishes (i.e. without reversing agent) received an equivalent amount of the appropriately diluted solvent. At the concentrations employed, the various reversing agents do not show any significant toxicity towards the cell lines. The dishes were incubated for 6–8 days at 37°C in a 5% $CO_2$/95% air incubator. Subsequently, the dishes were stained for about 30 min with 0.5% methylene blue in 50% methanol and the number of colonies in each well was scored. From the average numbers of colonies observed in the presence of different drug concentrations, the $D_{10}$ values (i.e. drug concentrations which reduced cloning efficiency to approximately 10% of that in the absence of any drug) of different cell lines in the absence and presence of various reversing agents were determined. The degree of resistance of any cell line was determined from the ratio of $D_{10}$ values for the mutant versus parental cell lines. The sensitising effect of reversing agents was calculated from the ratios of $D_{10}$ values observed in the absence and presence of reversing drug(s).

### Results

We examined the $pH_i$ of several multidrug resistant cell lines. Highly resistant $CH^RC5$ and DC3F/ADX cells maintained a $pH_i$ that was about $0.15 \pm 0.03$ pH units above that of the parental cell line. Cells with a lower degree of resistance showed less difference in $pH_i$ compared to their drug-sensitive counterparts (Table I). The higher values of $pH_i$ for the $CH^RC5$ resistant cell lines were observed regardless of extracellular pH ($pH_o$) (Figure 1).

Since a $Na^+/H^+$ exchange system could be involved in the control of $pH_i$ in these cell lines, we studied the ability of $AB_1$ and $CH^RC5$ cells to recover from an intracellular acid load after incubation with nigericin (Figure 2). The cytoplasmic alkalinisation was completely inhibited by 100 μM amiloride, indicating that a $Na^+/H^+$ exchange system is active and does play an important role in controlling the $pH_i$ in this cell line (Figure 2). Neither cyclosporin A nor verapamil had any effect on the rate of pH recovery after acid loading. The rate of recovery upon addition of NaCl was higher in the resistant than in the sensitive cells (Figure 3).

A number of drugs have been shown to sensitise multidrug resistant cells to cytotoxic agents. We measured the effects of several of these drugs on $pH_i$ and on the sensitivity of cells to the cytotoxic effect of vinblastine. We also tested the effects of amiloride, a known inhibitor of $Na^+/H^+$ antiport, on the reversal of multidrug resistance. This was done with parental and drug resistant CHO and CHL cells. As seen from Table II, treatment of either $AB_1$ or DC3F cells with either 5–20 μM verapamil or 3 μM cyclosporin A sensitises them by a factor of up to about 10-fold towards vinblastine. This sensitisation, as shown recently (Gupta, 1988), is due to the fact that Chinese hamster cells display an intrinsic MDR phenotype, in comparison to human cells, which are reversed by these agents. Verapamil at the above concentrations also caused a dose-dependent reversal of vinblastine resistance in the two mutant cell lines. At the higher concentration, the cells became nearly as sensitive as the parental line in the presence of verapamil. However, in contrast to verapamil, cyclosporin A was effective in sensitising only the $CH^RC5$

**Table I** $pH_i$ of MDR cells

| *Cell line* | *Reference* | *Cell type* | *Selecting drug* | *Relative drug resistance* | *$pH_i$*[a] *Parental cell line* | *$pH_i$*[a] *Resistant cell line* |
|---|---|---|---|---|---|---|
| $CH^RC5$ | Bech-Hansen *et al.* (1976) | CHO | Colchicine | 180 | 7.01±0.03 (9) | 7.18±0.03 (9) |
| DC3F/ADX | Biedler & Riehm (1970) | CHL | Actinoymcin D | 2,500 | 7.02±0.02 (6) | 7.16±0.03 (6) |
| HeLa $Pur^{RII-7}$ | Gupta *et al.* (1988) | Human | Puromycin | 50 | 6.95±0.02 (3) | 7.05±0.02 (3) |
| $Tax^{R-2}$ | Gupta (1983) | CHO | Taxol | 8 | 7.03±0.02 (2) | 7.07±0.03 (2) |
| KB-C1 | Akiyama *et al.* (1985) | Human | Colchicine | 260 | 6.96±0.02 (2) | 7.08±0.02 (2) |

[a]Intracellular pH was measured with the fluorescent probe BCECF as indicated in Materials and methods. Values are the means ± s.e.m. of several experiments (indicated in parentheses).

**Figure 1** $pH_i$ dependence on $pH_o$ in CHO cells. $pH_i$ as a function of $pH_o$ in a drug-sensitive cell line, $AB_1$ (○) and a drug-resistant cell line, $CH^RC5$ (●). The cells were pre-equilibrated in $HCO_3^-$-free media for 60 min at the indicated pH. Then the cells were loaded with BCECF/AM and the $pH_i$ was measured as indicated in Materials and methods. Each point is the mean of triplicate determinations. Error bars represent s.d.

**Figure 2** Measurement of $Na^+/H^+$ antiport activity. Cells were acidified with the addition of nigericin. Acidification was terminated with the addition of fatty acid-free albumin. $Na^+/H^+$ antiport activity was initiated with the addition of 50 mM NaCl (see Methods). Amiloride (200 μM) completely blocked the increase in $pH_i$ while addition of either cyclosporin A (20 μM) or verapamil (40 μM) with the NaCl had no effect on the recovery from acidification.

**Figure 3** $Na^+/H^+$ exchange in CHO cells. Relationship between the rate of $pH_i$ recovery, i.e. $Na^+/H^+$ exchange activity, and $pH_i$ in a drug sensitive cell line, $AB_1$ (○) and a multiple drug resistant cell line, $CH^RC5$ (●). $Na^+/H^+$ antiport activity was measured as indicated in Materials and methods. Each point is the average of triplicate determinations.

line but had no effect of DC3F/ADX cells. It is interesting to note that, although cyclosporin had no effect on the DC3F/ADX line, the parental cells were sensitised by a factor of about 10 in its presence. In contrast to these compounds, amiloride had no sensitising effect on any of the sensitive or resistant cell lines. Similar results with the above compounds for these cell lines have also been obtained for another drug (colchicine) to which the MDR mutants exhibits increased resistance (results not shown).

Table III shows the effect of the above compounds on intracellular pH in the two sets of sensitive and resistant cell lines. In the case of Cs A and verapamil, the cells were pre-incubated at 37°C for 30 min in the presence of the modifier before measuring $pH_i$ in the absence of modifier. When $pH_i$ was measured in the presence of verapamil, similar results were obtained. In the case of amiloride, the cells were not pre-incubated with drugs but amiloride was present during the measurement of $pH_i$. Cyclosporin A with the CHO cells is the only case where there is both intracellular acidification of the resistant cell line to the pH of the sensitive cell line and reversal of MDR.

### Discussion

We have shown that the $pH_i$ of a number of different multidrug resistant cell lines is higher than their parental counter-

**Table II** Effect of different agents on the relative drug resistance of various cell lines

| | *Relative resistance to vinblastine*[a] *(fold sensitisation)* | | | |
|---|---|---|---|---|
| *Compounds* | *$AB_1$* | *$CH^RC5$* | *DC3F* | *DC3F/ADX* |
| Control (no addition) | 1.0 | 50.0 | 1.0 | 3000 |
| + Cyclosporin A (3 μM) | 0.1 (10) | 0.15 (330) | 0.1 (10) | 3000 (1) |
| + Verapamil (4 mM) | 0.35 (2.9) | 1.2 (42) | 0.30 (33) | 5.5 (545) |
| (20 μM) | 0.1 (10) | 0.4 (125) | 0.1 (10) | 0.5 (6000) |
| + Amiloride (200 μM) | 1.0 (1) | 50.0 (1) | 1.0 (1) | 3000 (1) |

[a]The experiments were done as described in Materials and methods. Assuming the $D_{10}$ value of vinblastine for the parental sensitive cell lines ($AB_1$, 5 nM; DC3F, 3.5 nM) in the absence of any reversing agents to be 1, the relative resistance of the cell lines under different conditions are indicated. The numbers in parentheses show the fold sensitisation of the cell lines (as compared to the control lacking any sensitising drug) in the presence of indicated concentrations of the reversing agents. A fold sensitisation of 1 indicates no change in sensitisation. Similar results with these cell lines and agents have been obtained in at least two independent experiments.

**Table III** Effect of drugs on $pH_i$ and MDR

| *Modifier* | *$pH_i$* | *$pH_i$* | *Reversal MDR* |
|---|---|---|---|
| *Chinese hamster ovary cells* | | | |
| | *$AB_1$* | *$CH^RC5$* | |
| None | 7.01±0.03 | 7.18±0.03 | – |
| Cs A (20 μM) | 6.98±0.03 | 7.00±0.02 | Complete reversal |
| Verapamil (40 μM) | 6.97±0.02 | 7.15±0.03 | Complete reversal |
| Amiloride (200 μM) | 6.95±0.01 | 7.06±0.02 | No effect |
| *Chinese hamster lung cells* | | | |
| | *DC3F* | *DC3F/ADX* | |
| None | 7.02±0.02 | 7.16±0.03 | – |
| Cs A (20 μM) | 6.97±0.02 | 7.12±0.02 | No effect |
| Verapamil (40 μM) | 7.00±0.01 | 7.13±0.02 | Complete reversal |
| Amiloride (200 μM) | 6.93±0.02 | 7.08±0.03 | No effect |

Intracellular pH was measured with the fluorescent probe BCECF. Reversal of MDR indicates the ability of the modifier to sensitise the cell line to the cytotoxic action of vinblastine. Values are the mean ± s.e.m. of triplicate determinations. Cs A is cyclosporin A.

part. The magnitude of the difference between the resistant and sensitive cell lines is related to the degree of resistance, with the most resistant cell lines showing the greatest alkalinisation of intracellular pH (Table I). However, there is no direct proportionality between the degree of resistance and $pH_i$, which is in contrast to a previous report which showed a linear relationship between resistance and $pH_i$ for a series of increasingly multidrug resistant variants of a human lung tumour cell line (Keizer & Joenje, 1989). The lack of quantitative correlation between drug resistance and $pH_i$ for different cell lines does not rule out a role for $pH_i$ in resistance since there may be many differences among the different cell lines. However, as we will show below, there are a number of lines of evidence to demonstrate the lack of a consistant correlation between intracellular pH and multidrug resistance.

The observed increased activity of the $Na^+/H^+$ antiporter in one of the resistant cell lines (Figue 3) is consistent with the hypothesis that this antiport mechanism is responsible for the higher $pH_i$ found in resistant cells. However, blockage of this activity by amiloride does not reverse multidrug resistance (Table III). Of course the lack of effect of amiloride in the clonogenic assay is negative evidence and therefore not conclusive. It could be due, for example, to the metabolic instability of amiloride in the cell cultures used. In addition, however, cyclosporin A and verapamil, which reverse multidrug resistance, have no effect on $Na^+/H^+$ antiport activity. Therefore, the higher antiport activity observed in the $CH^RC5$ drug resistant cells does not appear to be closely associated with the mechansim of their resistance. There are a number of possible causes for the increased $Na^+/H^+$ antiport activity in resistant cells, including increased expression of the antiporter, alteration in the pH dependence of antiporter activity or changes in the regulation of antiporter activity. The $Na^+/H^+$ exchange activity is activated by protein kinase C (Siffert & Akkerman, 1988). Protein kinase C activity is higher in several but not all multidrug resistant cell lines (Palayoor *et al.*, 1987; Fine *et al.*, 1988). It is possible that the alkalinisation of multidrug resistant cell lines is an indirect manifestation of a higher protein kinase C activity. It is also possible that the increased $pH_i$ of multidrug resistant cells is not a result of changes in $Na^+/H^+$ antiport activity but rather to differences in metabolic activities between parental and resistant cell lines (Lyon *et al.*, 1988). Further studies are required to determine the generality of the changes in $Na^+/H^+$ antiport activity with multidrug resistance and to determine the cause of such changes. However, the changes in $Na^+/H^+$ antiport activity appear independent of the mechanism of drug resistance and their contribution to the higher $pH_i$ of resistant cells remains to be determined.

Although higher $pH_i$ appears to be a general characteristic of all MDR cell lines examined, its relevance to the MDR phenotype is at present unclear. Amiloride acidifies the $pH_i$ in both sensitive and resistant cell lines, but it does not cause any reversal of MDR. The pH difference between the parental and the resistant cell lines is maintained (although somewhat reduced in the case of CHO cells) even in the presence of amiloride (Table III), suggesting that $Na^+/H^+$ antiport may be less important for the maintenance of a higher pH in the resistant cells. Further, if the higher $pH_i$ in the resistant cells was related to their MDR phenotype, then treatment with agents which cause reversal of the MDR phenotype should abolish the $pH_i$ difference between sensitive and resistant cell line. However, such a correlation was not observed for verapamil, which caused complete reversal of vinblastine resistance in the two sets of cell lines without changing their $pH_i$. In the study of Keizer and Joenje (1989) verapamil did lower the $pH_i$ of resistant cells at concentrations greater than 4 $\mu$M. The cell lines used in that work had particularly high $pH_i$ values for their degree of resistance and they showed greater acidification by verapamil than the resistant clones used in the present work. The origin of these differences is not known but it is clear that acidification of resistant cells is not a general property of verapamil. Furthermore, both the $Na^+/H^+$ ionophore, monensin, which would increase $pH_i$, and the $K^+/H^+$ ionophore, nigericin, which would decrease $pH_i$, increase drug accumulation in resistant cells (Sehested *et al.*, 1988). This is another indication that there is no correlation between $pH_i$ and cell resistance. In contrast to verapamil, interesting results were obtained with cyclosporin A. It was observed that the concentrations of cyclosporin which completely reversed vinblastine resistance in $CH^RC5$ cells had *no* observable effect on the DC3F/ADX cells (although it sensitised the parental DC3F cells by a factor of about 10). To our knowledge, this is the first report where such marked specificity (or differences) towards a reversing agent has been observed between two MDR cell lines. The observed difference between $CH^RC5$ and DC3F/ADX cell in their response to cyclosporin A points to some important difference in the mechanisms leading to MDR phenotype in the two cell lines. Interestingly, and in contrast to verapamil, the reversal of drug resistance in $CH^RC5$ by cyclosporin A was accompanied by a lowering of $pH_i$ to the same level as the sensitive $AB_1$ cells. However, cyclosporin A had no effect on the $pH_i$ of the DC3F/ADX cells. It thus appears that the reversal of multidrug resistance by cyclosporin A is closely associated with a process which causes a lowering of $pH_i$. This process is not the inhibition of $Na^+/H^+$ antiport since we have shown that cyclosporin has no effect on this mechanism. However, the lowering of $pH_i$ by cyclosporin is a collateral event, rather than being the mechanism of reversal of drug resistance by this agent. Further investigation of the mechanism by which cyclosporin A causes reversal of the MDR phenotype and affects $pH_i$ and the manner in which the two MDR cell lines examined differ should be of considerable interest.

We are grateful to Drs Victor Ling, June Biedler and Michael Gottesman for providing the cell lines which were used in this work. The cyclosporin A was kindly provided by Sandoz Pharmaceutical Co. This work was supported by grants from the National Cancer Institute of Canada and from the Medical Research Council.

### References

AKIYAMA, S.I., FOJO, A., HANOVER, J.A., PASTAN, I. & GOTTESMAN, M.N. (1985). Isolation and genetic characterization of human KB cell lines resistant to multiple drugs. *Somat. Cell Mol. Genet.*, **11**, 117.

BECH-HANSEN, N.T., TILL, J.E. & LING, V. (1976). Pleiotropic phenotype of colchicine-resistant CHO cells: cross-resistance and collateral sensitivity. *J. Cell Physiol.*, **88**, 23.

BIEDLER, J.L. & RIEHM, H. (1970). Cellular resistance to actinomycin D in Chinese hamster cells *in vitro*: cross-resistance, radioautographic and cytogenetic studies. *Cancer Res.*, **30**, 1174.

BRADLEY, G., JURANKA, P.F. & LING, V. (1988). Mechanism of multidrug resistance. *Biochim. Biophys. Acta*, **948**, 87.

DANØ, K. (1973). Active outward transport of daunomycin in resistant Erlich ascites tumor cells. *Biochim. Biophys. Acta*, **323**, 466.

FINE, R.L., PATEL, J., CARMICHAEL, J., COWAN, K.H. & CHABNER, B.A. (1988). Phosphoprotein and protein kinase C changes in human multidrug-resistant cancer cells. In *Mechanisms of Drug Resistance in Neoplastic Cells*, Woolley, P.V. III & Tew, K.D. (eds) p. 87. Academic Press: San Diego, CA.

GERLACH, J.H., KARTNER, N., BELL, D.R. & LING, V. (1986). Multidrug resistance. *Cancer Surveys*, **5**, 25.

GRINSTEIN, S. (1988). The intracellular pH of white blood cells: measurement and regulation. *Biochem. Cell Biol.*, **66**, 245.

GRINSTEIN, S., COHEN, S. & ROTHSTEIN, A. (1984). Cytoplasmic pH regulation in thymic lymphocytes by an amiloride-sensitive $Na^+/H^+$ antiport. *J. Gen. Physiol.*, **83**, 341.

GUPTA, R.S. (1983). Taxol resistant mutants of Chinese hamster ovary cells: genetic, biochemical and cross resistance studies. *J. Cell Physiol.*, **114**, 137.

GUPTA, R.S. (1988). Intrinsic multidrug resistant phenotype of Chinese hamster (rodent) cells in comparison to human cells. *Biochem. Biophys. Res. Commun.*, **153**, 598.

GUPTA, R.S., MURRAY, W. & GUPTA, R. (1988). Cross resistance pattern toward anticancer drugs of a human carcinoma multidrug-resistant cell line. *Br. J. Cancer*, **58**, 441.

INABA, K., WATAMABE, T. & SUGIYAMA, Y. (1987). Kinetic analysis of active efflux of vincristine from multidrug resistant P388 Leukemia cells. *Jpn. J. Cancer Res. (Gann)*, **78**, 397.

KEIZER, H.G. & JOENJE, H. (1989). Increased cytosolic pH in multidrug resistance human lung tumor cells: effect of verapamil. *J. Natl Cancer Inst.*, **81**, 706.

LYON, R.C., COHEN, J.S., FAUSTINO, P.J., MEGNIN, F. & MYERS, C.E. (1988). Glucose metabolism in drug-sensitive and drug-resistant human breast cancer cells monitored by magnetic resonance spectroscopy. *Cancer Res.*, **48**, 870.

PALAYOOR, S.T., STEIN, J.M. & HAIT, W.N. (1987). Inhibition of protein kinase C by antineoplastic agents: implications for drug resistance. *Biochem. Biophys. Res. Commun.*, **148**, 718.

RINK, T.J., TSIEN, R.Y. & POZZAN, T. (1982). Cytoplasmic pH and free $Mg^{2+}$ in lymphocytes. *J. Cell Biol.*, **95**, 189.

SEHESTED, M., SKOVSGAARD, T. & ROED, H. (1988). The carboxylic ionophore monenin inhibits active drug efflux and modulates *in vitro* resistance in daunorubicin resistant Ehrlich ascites tumor cells. *Biochem. Pharmacol.*, **37**, 3305.

SIFFERT, W. & AKKERMAN, J.W.N. (1988). Protein kinase C enhances $Ca^{2+}$ mobilization in human platelets by activating $Na^+/H^+$ exchange. *J. Biol. Chem.*, **263**, 4223.

THOMAS, J.A., BUCHSBAUM, R.N., ZIMNIAK, A. & RACKER, E. (1979). Intracelluar pH measurements in Ehrlich ascites tumor cells utilizing spectroscopic probes generated *in situ*. *Biochemistry*, **18**, 2210.